# Comparison of transabdominal ultrasound and electromagnetic transponders for prostate localization

**DOI:** 10.1120/jacmp.v11i1.2924

**Published:** 2010-01-06

**Authors:** Ryan D. Foster, Timothy D. Solberg, Haisen S. Li, Andrew Kerkhoff, Charles A. Enke, Twyla R. Willoughby, Patrick A. Kupelian

**Affiliations:** ^1^ Department of Radiation Oncology UT Southwestern Medical Center at Dallas Dallas TX USA; ^2^ Department of Radiation Oncology University of Pittsburgh Medical Center at Shadyside Pittsburgh PA USA; ^3^ London School of Hygiene and Tropical Medicine London United Kingdom; ^4^ Department of Radiation Oncology University of Nebraska Medical Center Omaha NE USA; ^5^ Department of Radiation Oncology M. D. Anderson Cancer Center Orlando Orlando FL USA

**Keywords:** Localization, Prostate, Ultrasound, electromagnetic transponders

## Abstract

The aim of this study is to compare two methodologies of prostate localization in a large cohort of patients. Daily prostate localization using B‐mode ultrasound has been performed at the Nebraska Medical Center since 2000. More recently, a technology using electromagnetic transponders implanted within the prostate was introduced into our clinic (Calypso). With each technology, patients were localized initially using skin marks. Localization error distributions were determined from offsets between the initial setup positions and those determined by ultrasound or Calypso. Ultrasound localization data was summarized from 16,619 imaging sessions spanning seven years. Calypso localization data consists of 1524 fractions in 41 prostate patients treated in the course of a clinical trial at five institutions and 640 localizations from the first 16 patients treated with our clinical system. Ultrasound and Calypso patients treated between March and September 2007 at the Nebraska Medical Center were analyzed and compared, allowing a single institutional comparison of the two technologies. In this group of patients, the isocenter determined by ultrasound‐based localization is on average 5.3 mm posterior to that determined by Calypso, while the systematic and random errors and PTV margins calculated from the ultrasound localizations were 3–4 times smaller than those calculated from the Calypso localizations.

Our study finds that there are systematic differences between Calypso and ultrasound for prostate localization.

PACS number: 87.63.dh

## I. INTRODUCTION

One of the greatest challenges in radiation oncology is the uncertainty of tumor and organ position inside the patient. Computed tomography (CT) scans used for treatment planning are snapshots of the patient taken days before treatment begins. In the case of prostate cancer, variable filling of the bladder and rectum virtually guarantee that on the first day of treatment the target anatomy will not be in the same position as the day of the planning CT scan. To account for setup uncertainty and organ motion, the ICRU^(^ ^1^ ^,^ ^2^ ^)^ has recommended that a margin be added to the target during the planning process. Unfortunately, the planning target volume (PTV) often includes healthy tissues or organs that are irradiated unnecessarily. If the prostate could be localized more accurately, the margin could be reduced and more healthy tissue could be spared. Accurate localization is even more critical for dose escalation and hypofractionation.

Prostate localization has been studied extensively and several different technologies have been used for daily localization, including transabdominal ultrasound, X‐ray portal imaging, and kilovoltage and megavoltage cone beam CT.^(^ ^3^ ^–^ ^8^ ^)^ With these technologies, patients are localized using bony anatomy, implanted fiducials, or three‐dimensional volumetric images. These image‐guided technologies have allowed the therapist to determine the magnitude and direction of the setup error and correct for it before treatment is initiated. Numerous studies have been published describing the merits of the various methods; in general, localization uncertainties are on the order of 0.5 cm (1σ) in each direction.^(^ ^8^ ^–^ ^18^ ^)^ While each of these localization techniques has limitations, ultrasound has emerged as a common prostate localization methodology in the radiotherapy community.^(^ ^19^ ^–^ ^23^ ^)^ Ultrasound is particularly attractive in that it is noninvasive and does not use ionizing radiation.

Despite its popularity, the accuracy of ultrasound localization of the prostate has been questioned. Scarbrough et al.^(12)^ found that ultrasound localization differs from kV localization of implanted markers by an average three‐dimensional distance of 8.8 mm. Other studies^(^ ^21^ ^,^ ^24^ ^)^ have found poor agreement between ultrasound and X‐ray imaging of implanted markers. Also, ultrasound can be subjective since localization involves visual alignment of contours based on CT from the treatment planning system to the respective organs on the ultrasound images. Different users may not agree on what constitutes a good alignment, and the ultrasound images depend heavily on the technique used to acquire them. The angle of the probe and the amount of pressure used influence the quality of the images. The pressure on the patient's abdomen may introduce a systematic error in the prostate localization.^(^ ^25^ ^,^ ^26^ ^)^ It has been shown^(21)^ that the interuser variability of ultrasound localization can be significant.

The Calypso 4D localization system (Calypso Medical Technologies, Inc., Seattle, WA) is the first localization technology to provide completely objective localization capabilities with continuous monitoring of the prostate position during radiation therapy. By utilizing electromagnetic detection of three transponders implanted in the prostate, Calypso provides a method of localizing prostate patients that, in contrast to ultrasound or cone‐beam CT, does not involve visual alignment of a pair of images or contours.

The purpose of this study is to compare ultrasound and Calypso for localization of the prostate in a large population of patients. While we present localization data collected from several different institutions, the manuscript emphasizes the comparison and analysis of localization data from a single institution for patients treated during the same time period. This paper investigates the strengths and weaknesses of the two localization technologies and seeks to determine if there is any benefit in using Calypso over ultrasound.

## II. MATERIALS AND METHODS

**Figure 1:**
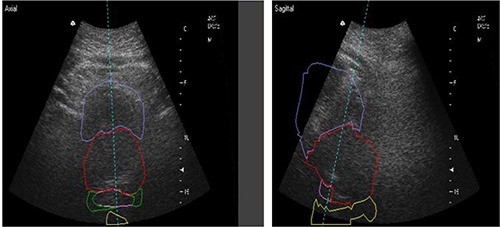
Axial and sagittal ultrasound images shown superimposed with bladder, prostate, seminal vesicles, and rectum contours from the treatment planning CT.

Ultrasound localization requires the acquisition of two ultrasound images (axial and sagittal) to which the patient's treatment planning contours are aligned (Fig. 1). Once the user has aligned the contours to the ultrasound images, the software calculates the respective couch shifts from the initial set‐up position (determined from skin marks) based on the contour‐image alignment.

The B‐mode Acquisition and Targeting (BAT) ultrasound system (NOMOS, Sewickley, PA) has been used at the Nebraska Medical Center since 2000 for daily prostate localization. The ultrasound data presented here have been collected from 16,619 treatment sessions. Localization error distributions have been determined from offsets between the initial setup positions and those determined by ultrasound.

**Figure 2:**
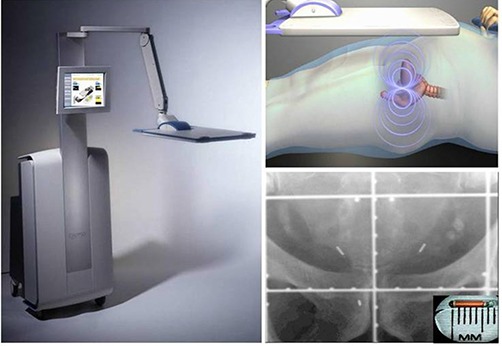
The Calypso console (left) with touch screen monitor and transmitter / receiver array, which generates a resonant signal in 8mmlong×2mm diameter transponders (top‐right) implanted in the right and left base and the apex of the prostate (bottom‐right). The implantation is done under the guidance of ultrasound in a manner similar to a needle biopsy.

The Calypso system uses an array of AC magnetic coils to generate a resonant response from implanted electromagnetic transponders (Beacons), which is detected by a second array of receiver coils (Fig. 2). The array position relative to the linac isocenter is determined by three infrared cameras mounted on the ceiling in the room. Daily quality assurance is performed each morning and the entire system is calibrated monthly. The Beacons (8 mm in length and 2 mm in diameter) are implanted in the right and left base and the apex of the prostate. The implantation is performed under transrectal ultrasound guidance. The coordinates of the Beacons and the isocenter are identified on the treatment planning CT and entered into the Calypso tracking station. Similar to ultrasound, initial patient localization is performed using skin marks to align with room lasers. Calypso is used to localize the patient and the system calculates the initial offset. The couch is shifted until the three offsets are zero. During treatment, Calypso monitors and reports the offset between the actual and planned isocenter position at a rate of 10 Hz.

Calypso has been shown to have submillimeter accuracy.^(27)^ Additionally, Calypso localization compares favorably to that using kV X‐ray localization of implanted radio‐opaque markers,^(28)^ with a positional stability of the transponders that is similar to that of gold seeds.^(29)^

Calypso localization data has been collected from a multi‐institution clinical trial in 2006 and from sixteen patients treated at NMC from March through September 2007 on our clinical system. Localization error distributions were calculated from the setup offsets as determined by Calypso.

We present seven years of ultrasound localization data at NMC and the Calypso data from the clinical trial, but since these are clearly different patient populations, we do not compare the localization results from these two groups. We do compare the ultrasound and Calypso data from patients treated in 2007 at NMC and perform statistical analysis on these two groups.

For the patients localized at our institution from March through September 2007, a t‐test was performed to determine if the means of the distributions are significantly different and the F‐test was used to analyze differences in the variances of the shift distributions. The ultrasound and Calypso data were also analyzed using the Kolmogorov‐Smirnov (K‐S) test to determine whether or not the two datasets came from the same continuous distribution. P values<0.01 were considered statistically significant. MATLAB version 7.8 was used for statistical analysis. For each localization method, the systematic error Σ for the population was calculated by taking the standard deviation of the individual patient means and the random error σ was determined by calculating the root mean square of the individual patient standard deviations. The PTV margins necessary to account for these errors was determined using the Van Herk^(30)^ formula:

(1) M=2 Σ+0.7 σ

## III. RESULTS

**Figure 3:**
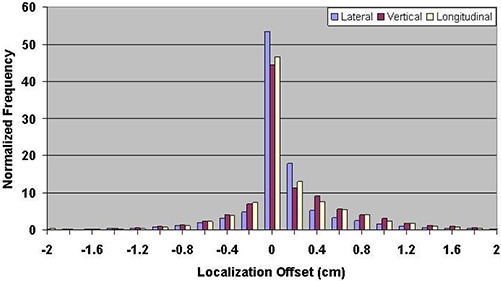
Distribution of setup errors relative to skin marks in 16,619 ultrasound localization procedures performed from 2000 through 2007.

**Figure 4:**
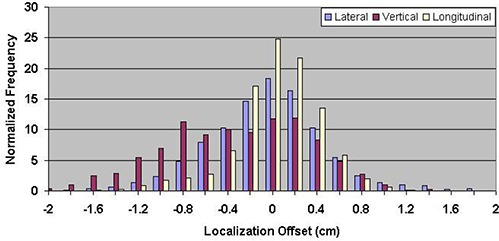
Distribution of setup errors relative to skin marks in 1524 Calypso localization procedures performed during a multi‐institutional clinical trial.

Table 1 shows the mean errors relative to skin marks for 16,619 ultrasound localization procedures in patients treated from 2000 through 2007, and 1524 Calypso localization procedures in patients treated in the multi‐institutional clinical trial. The mean localization errors as determined by ultrasound were 0.17±3.80mm, −0.66±4.34 mm, and 0.76±4.75mm in the lateral, vertical, and longitudinal directions, respectively, where positive localization errors represent posterior, right (as viewed from the foot of the table), and superior couch shifts. The corresponding setup errors determined by Calypso were −1.26±5.64 mm, −4.26±6.37 mm, and −0.73±3.77 mm, respectively. Figures 3 and 4 contain the localization error distributions for the ultrasound and Calypso patients, respectively.

**Table 1:** Mean localization errors relative to skin marks for all ultrasound patients (2000–2007) and Calypso patients from the clinical trial.

	*Number of Localization Procedures*	*Lateral (mm)*	*Vertical (mm)*	*Longitudinal (mm)*
Calypso	640	−1.26±5.64	−4.26±6.37	−0.73±3.77
Ultrasound	829	0.17±3.80	−0.66±4.34	0.76±4.75

**Figure 5:**
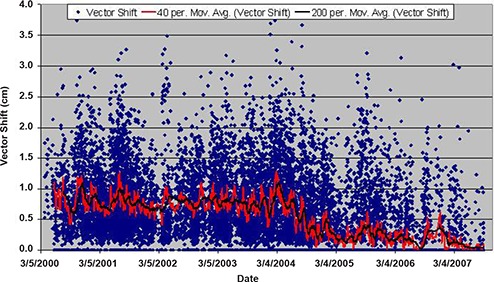
Moving average of the 3D vector shift for patients localized with BAT in chronological order from 2000 through September 2007. For clarity, a 40 and 200 point moving average is shown in the red and black lines, respectively. Note the change that occurred in March 2004 due to a modification in the ultrasound localization procedure.

**Figure 6:**
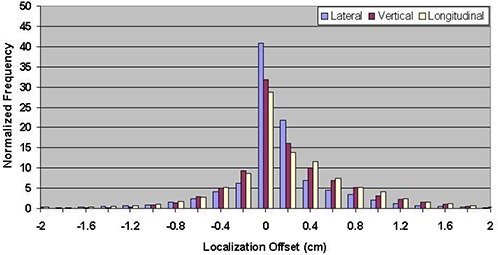
Distribution of setup errors relative to skin marks in 10,913 ultrasound localization procedures performed from 2000 through 2004.

**Figure 7:**
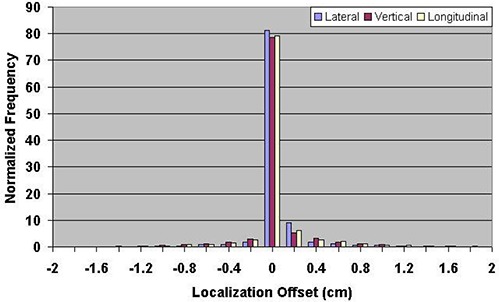
Distribution of setup errors relative to skin marks in 5706 ultrasound localization procedures performed from 2005 through 2007.

During the course of the study, it was noted that the technique for ultrasound localization at our institution was modified in 2004. The ultrasound probe can introduce a posterior shift of the prostate, so less pressure was applied to the patient's abdomen starting in 2004. Figure 5 shows the vector displacement of all ultrasound localization procedures as a function of time. The procedural change in BAT localization that occurred in 2004 is apparent as the vector displacement is reduced by a factor of two, beginning in approximately March 2004. Figures 6 and 7 show the ultrasound localization error distributions for the time periods 2000 through 2004 and 2005 through 2007. Again, there is a clear difference, with the latter period highly peaked about zero.

**Figure 8:**
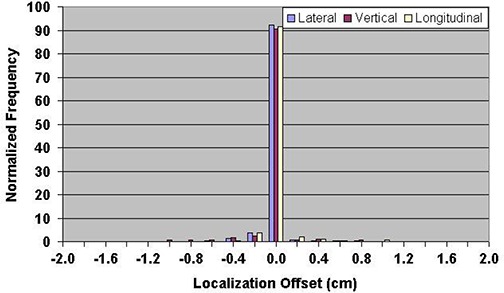
Distribution of setup errors relative to skin marks in 829 ultrasound localization procedures performed between March and September, 2007.

**Figure 9:**
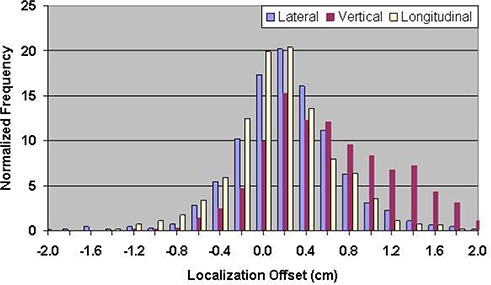
Distribution of setup errors relative to skin marks in 640 Calypso localization procedures performed between March and September, 2007.

To address potential procedural differences among the five participating clinical trial sites, a retrospective study was performed by comparing the Calypso and BAT patients treated only at a single institution in 2007. In this case, the couch shifts observed in the lateral, vertical, and longitudinal directions were 1.29±5.17mm, 5.19±6.24mm, 0.66±4.81mm (Calypso) and −0.04±2.98 mm, −0.10±3.34 mm, and 0.23±6.14mm (BAT), respectively. The mean localization errors can be found in Table 2. In this group of patients, the isocenter determined by ultrasound‐based localization is on average 5.3 mm posterior to that determined by Calypso. The localization error distributions for ultrasound and Calypso patients treated between March and September 2007 are shown in Figs. 8 and 9, respectively.

**Table 2:** Mean localization errors relative to skin marks for ultrasound and Calypso for patients treated 3/07–9/07.

	*Number of Localization Procedures*	*Lateral (mm)*	*Vertical (mm)*	*Longitudinal (mm)*
Calypso	640	1.29±5.17	5.19±6.24	0.66±4.81
Ultrasound	829	−0.04±2.98	−0.10±3.34	0.23±6.14

Table 3 contains the *p* values for the Kolmogorov‐Smirnov, the t‐, and the F‐tests following a statistical analysis of the patients treated March – September 2007 at NMC. The hypothesis that the Calypso and ultrasound data came from the same distribution is rejected. The t‐test shows that the means of the shift distributions are significantly different and the F‐test shows that the variances are not equal. The systematic and random errors for the two localization methods are in Table 4, along with the calculated PTV margins.

**Table 3:** P values for the Kolmogorov‐Smirnov, t‐, and F‐tests performed on ultrasound and Calypso setup error distributions from patients treated 3/07– /07.

*Test*	*Lateral*	*Vertical*	*Longitudinal*
Kolmogorov‐Smirnov	<0.001	<0.001	<0.001
t‐test	<0.001	<0.001	0.0015
F‐test	<0.001	<0.001	<0.001

**Table 4:** Systematic error, random error, and PTV margins for patients localized with ultrasound and Calypso at NMC 3/07–9/07.

	*Lateral (mm)*	*Ultrasound Vertical (mm)*	*Longitudinal (mm)*	*Lateral (mm)*	*Calypso Vertical (mm)*	*Longitudinal (mm)*
Systematic	0.59	1.31	1.03	2.75	4.87	3.76
Random	1.63	1.35	1.20	4.65	4.55	3.03
PTV margin	2.32	3.57	2.9	8.76	12.93	9.63

## IV. DISCUSSION

The ultrasound error distributions are reasonably symmetric about zero, with a standard deviation that is consistent with data reported previously in the literature.^(^ ^19^ ^–^ ^23^ ^)^ The ultrasound error distributions are somewhat narrower than the Calypso distributions, suggesting there is more uncertainty in prostate localization than the ultrasound data alone would suggest. This is understandable, given the subjective nature of the procedure, the difficulty visualizing the prostate, and interuser variations. The mean vertical couch shifts from the five institutions that participated in the clinical trial were in the anterior direction and ranged from 2.6 to 8.2 mm. The average ultrasound localization does not exhibit such a large vertical displacement.

The technique for ultrasound localization at NMC was modified in 2004 due to concern that pressure from the ultrasound probe might introduce a systematic error in the localization in the AP direction. This resulted in a significant reduction in the magnitude of shifts made relative to skin marks (Fig. 5) and a significant narrowing of the error distributions (Figs. 6 and 7). Clearly there is a trade‐off to be made with regard to pressure applied to the probe; if too much pressure is applied, the act of making the measurement alone may impact the result.^(^ ^25^ ^,^ ^26^ ^)^ In contrast, too little pressure can produce a suboptimal image that is difficult to interpret.

The 2007 data allow a more straightforward comparison between Calypso and ultrasound. This dataset consists of 829 ultrasound and 640 Calypso localizations. While a high clinical load prevented us from performing ultrasound and Calypso localizations on the same patients, similar population studies have been performed^(^ ^11^ ^,^ ^17^ ^,^ ^18^ ^)^ that provide a meaningful comparison and support our methodology. All of the patients in our analysis were imaged on the same CT scanner and treated on the same linac with the same immobilization techniques, and the localizations were performed by the same personnel. This is significant since any systematic errors due to differences in couch sag between the CT scanner and the linac or laser misalignments will be present in both sets of data. Daily and monthly quality assurance tests were performed on each localization system according to manufacturer's recommendations. The only difference between the two sets of patient data is the method used to localize them.

Despite the identical nature of the patient immobilization, setup, and treatment, the K‐S test showed that the two patient groups treated in 2007 do not belong to the same larger population, perhaps indicating that there are inherent differences in the localization methods. If the two localization methods were equal, we would expect similar distributions, means, and standard deviations of the one‐dimensional localization error distributions. The t‐ and F‐tests showed a significant difference (p<0.01) in the means and variances. The PTV margins necessary for patients localized with Calypso are 3–4 times larger than for those localized with ultrasound.

Mean one‐dimensional shifts that are greater than 1 mm represent clinically important systematic errors^(31)^ because one‐dimensional patient localization errors should be distributed symmetrically about zero. Due to the very small mean offset for the ultrasound patients, it appears that there is no systematic error present. This contradicts the majority of published ultrasound localization studies. However, when compared with the mean Calypso localization errors, it is apparent that systematic errors do exist, particularly in the vertical direction. These errors should be present in both datasets. Distributions of the localization errors reveal that the one‐dimensional ultrasound offsets are almost entirely zero and clearly different from those obtained through localization with the Calypso system. This could be a result of poor visualization of the prostate due to the technique used and explains why the PTV margins calculated from the ultrasound data are much smaller than those for Calypso. The lower image quality that resulted from the new ultrasound technique may have prevented the users from making any shifts. Our ultrasound PTV margins are significantly smaller than those published in the literature^(^ ^10^ ^,^ ^15^ ^,^ ^17^ ^,^ ^18^ ^,^ ^22^ ^–^ ^24^ ^)^ for prostate localization, while the Calypso PTV margins are similar to other prostate localization studies.

We observed that the mean vertical offset for Calypso patients treated at NMC in 2007 is in the opposite direction from the Calypso patients in the multi‐institutional clinical trial. Several factors contribute to this difference. During the clinical trial, the five institutions used different immobilization and setup devices, and bladder, rectal and dietary guidelines varied.^(28)^ All of the Calypso patients at NMC from 2007 were given the same set of dietary instructions and were immobilized using the same techniques. Also, the difference in couch sag between the Novalis linac used in the clinical trial and the Siemens linac used in 2007, along with differences in CT/treatment room laser misalignments contribute to the directional difference.

## V. CONCLUSIONS

Ultrasound localization of the prostate has been widely used in radiation oncology and is convenient for the patient because it does not require the implantation of markers in the prostate. Calypso uses electromagnetic tracking of implanted transponders to localize and track the prostate during radiation therapy. A comparison of the single institution ultrasound and Calypso localization data from 2007 clearly shows that the setup error distributions are different for these two technologies. PTV margins calculated from the ultrasound data are 3–4 times smaller than suggested from other prostate localization studies, while the margins determined from the Calypso data are similar to those observed in the literature. We attribute these differences to a difficulty in visualizing the prostate due to the technique used for the ultrasound imaging. Changing the ultrasound technique in 2004 clearly changed the localization results. On the other hand, Calypso has the advantage of providing objective localization information.
